# Psychological Distress, Post‐Traumatic Stress and Emotional Suppression in a Pregnancy After a Perinatal Death: A Longitudinal Survey

**DOI:** 10.1111/1471-0528.18212

**Published:** 2025-05-13

**Authors:** Joanna Beaumont, Debbie Smith, Emilie Bailey, Rebecca Barron, Emma Tomlinson, Alexander E. P. Heazell

**Affiliations:** ^1^ Division of Developmental Biology and Medicine, Maternal and Fetal Health Research Centre, Faculty of Biology, Medicine and Health, School of Medical Sciences University of Manchester Manchester UK; ^2^ Division of Psychology & Mental Health, Faculty of Biology, Medicine and Health, School of Health Sciences University of Manchester Manchester UK; ^3^ Saint Mary's Hospital Manchester University NHS Foundation Trust Manchester UK

**Keywords:** anxiety, depression, post‐traumatic stress, pregnancy after loss

## Abstract

**Objective:**

To evaluate parents' psychological distress and emotional suppression in the antenatal and postnatal periods of a pregnancy following a perinatal death.

**Design:**

Questionnaire.

**Setting:**

Tertiary Maternity Unit in the UK.

**Sample:**

Parents who were pregnant and attending a specialist antenatal clinic for pregnancy after loss.

**Methods:**

Partners and mothers completed questionnaire measures which evaluated their levels of depression, anxiety, post‐traumatic stress (PTS) and emotional suppression at 23 and 32 weeks' gestation, and 6 weeks postnatally. Repeated measures ANOVA or Friedman tests were used to identify significant changes in scores. Independent samples *t*‐tests or Mann–Whitney *U* tests were used to determine significant differences in partners' and mothers' group scores. The proportion of partners and mothers scoring above threshold for each measure was identified, and a one‐sample *t*‐test examined partners' and mothers' within‐couple scores.

**Main Outcome Measures:**

Psychological distress and emotional suppression.

**Results:**

Fifty‐one partners and 54 mothers completed the questionnaire. Partners', but not mothers', depression symptoms significantly decreased (*p* = 0.004; 95% CI 0.6–2.7) from 23 weeks' gestation (*M* = 11.32, SD = 5.35) to 32 weeks' gestation (*M* = 9.68, SD = 4.68). Mothers' depression symptoms significantly decreased (*z =* −3.91, *p* < 0.001) from 32 weeks gestation (median value of 13; IQR 8–17) to 6 weeks postnatal (median value of 10; IQR 5–13). Parents' anxiety levels did not change across the course of pregnancy. Mothers', but not partners', anxiety symptoms significantly decreased (*z =* −2.49, *p =* 0.013) from 32 weeks' gestation (median value of 7.5; IQR 4–14) to 6 weeks postnatal (median value of 6; IQR 2–13). PTS did not change across the course of a pregnancy, and mothers' symptoms continued into the postnatal period. Partners are more likely to hide their emotions during pregnancy than mothers (*z* = 3.35, *p* < 0.001).

**Conclusion:**

Parents who have experienced a prior perinatal death are likely to experience symptoms of anxiety, depression and PTS in a subsequent pregnancy. Anxiety in partners and PTS in mothers may continue into the postnatal period. Specialist mental health support (e.g., counselling) offered within a dedicated pregnancy after loss service is one way to support parents. Further research is needed to determine whether psychological distress impacts negatively on parent–child bonding.

## 1 Introduction

In 2022, 4604 babies were stillborn or died in the neonatal period in the UK [1]. Parents who have experienced a perinatal death are more likely to develop symptoms of anxiety, depression and post‐traumatic stress (PTS) than parents who have had live births [2, 3]. However, mothers' and partners' experiences of baby loss may differ. Partners have been found to hide their feelings after pregnancy loss to take on the role of the supporter for the birthing mother, potentially having negative consequences for their wellbeing [4, 5, 6, 7, 8, 9]; they also report feeling isolated in comparison to mothers whose suffering is more noticeable [5] and feel overlooked by healthcare professionals [10].

In the subsequent year following a loss, 63% of mothers will become pregnant again [11]. Mothers encounter many challenges during subsequent pregnancies such as high levels of anxiety, depression and grief [12, 13]. Much less is known about partners' experiences. However, in our earlier qualitative study, mothers expressed concern that their partners had little or no support [14]. In a subsequent pregnancy, baby loss may have greater associations with anxiety and depression in partners compared to mothers [15]. In addition, partners have been found to have significantly higher levels of anxiety and PTS antenatally in a subsequent pregnancy compared to matched controls [16]. However, partners' and mothers' anxiety, depression and PTS symptoms decline throughout pregnancy, but moderate PTS symptoms have remained at 8 months postnatal [17].

There are very few studies which have evaluated bereaved parents' psychological wellbeing following the birth of a live baby. This study aimed to expand current evidence to describe partners' and mothers' depression, anxiety, PTS and emotional suppression in the antenatal and postnatal periods of a pregnancy after loss. It also aimed to identify differences and similarities in levels of psychological distress and emotional suppression between partners and mothers. We hypothesised that partners would have lower levels of psychological distress and PTS, and higher levels of emotional suppression, than mothers. In addition, we expected partners' and mothers' anxiety, depression and PTS to change across the course of the pregnancy and into the postnatal period.

## 2 Methods

### 2.1 Design

Participants were recruited from a specialist clinic, the Rainbow Clinic, which is based in a tertiary hospital in the North‐West of England. Recruitment was from October 2022 to September 2023. The Rainbow Clinic provides maternity care for mothers who have experienced previous perinatal death or second‐trimester pregnancy loss. The model of care at the clinic follows the international consensus statement for care in pregnancies after stillbirth [18] which is recommended to be used as a basis for care [19]. Our previous study that quantified mothers' anxiety and depression scores in pregnancy after stillbirth [13] found that anxiety measured by GAD‐7 fell from 7 to 5 points (with a standard deviation of 3 points). For 80% power to detect this difference in participants in our study, 36 partners and 36 mothers would be required at each time point. Assuming a 40% loss to follow‐up, we aimed to recruit 60 partners and 60 mothers at 15 weeks' gestation to complete the questionnaire. Partners and mothers were eligible for inclusion if they were pregnant and attending the Rainbow Clinic after a prior stillbirth, neonatal death, or late termination of pregnancy (≤ 16 weeks). Partners and mothers were excluded if they were less than 16 years of age, lacked the capacity to consent, or were unable to read English.

### 2.2 Procedure

The study was given a favourable ethical opinion by the Health Research Authority, South Central–Berkshire B Research Ethics Committee (Ref 22/SC/0108). Potential participants were approached prior to or at their initial Rainbow Clinic appointment; they were given an information sheet and those who agreed to participate gave their informed signed consent. The information sheet stated that participants' data would be kept confidential; however, if researchers believed that the participant was at risk of harming themselves (e.g., through highly endorsing item 10 on the EPDS scale which relates to suicidal ideation) researchers would need to break confidentiality to put them in touch with relevant support (e.g., GP/care team/midwife). The information sheet also detailed four signposts to relevant services (e.g., midwife at the specialist clinic, baby loss charity) for participants to contact if they were experiencing symptoms of emotional distress and needed support.

Participants were sent a link to complete an online questionnaire at approximately 23 weeks' gestation, 32 weeks' gestation and 6 weeks after the birth of their baby. All of the questionnaires were in English. The questionnaire was administered via University of Manchester REDCap (Research Electronic Data Capture) and it contained the Edinburgh Postnatal Depression Scale (EPDS) [20], the Generalised Anxiety Disorder Questionnaire (GAD‐7) [21], the Impact of Event Scale (IES‐R) [22] and the 4‐item Suppression Scale from the Emotion Regulation Questionnaire (ERQ) [23] which measured the tendency to hide the behavioural expression of emotion. Four items measured the use of expressive suppression (‘I keep my emotions to myself’, ‘When I am feeling positive emotions, I am careful not to express them’, ‘I control my emotions by not expressing them’ and ‘When I am feeling negative emotions, I make sure not to express them’). Participants rated their responses on a 5‐point Likert scale (1 = strongly disagree to 5 = strongly agree).

We chose to use the EPDS as it has been validated for use with mothers in the antenatal [24, 25] and the postnatal period [20], showing good reliability (*α*'s = 0.82–0.87) [20, 24]. It has also been validated for use with fathers showing good internal consistency (*α* = 0.81) [26]. The GAD‐7 scale was used as it is a recommended screening measure for perinatal anxiety by NICE [27] and has shown good psychometric properties when used with pregnant women [28, 29, 30, 31] including very good internal consistency (*α*'s = 0.89–0.91) [28, 30, 31]. It has also been shown to be a reliable and valid measure of anxiety in men (*α* = 0.89) [32]. We also used the EPDS and GAD‐7 scales in our previous work with mothers to measure symptoms of postnatal depression and anxiety [13]; using the scales in this study would allow us to identify whether rates of postnatal depression and anxiety in mothers experiencing a pregnancy after perinatal death have changed since 2016–18.

We chose to use the IES‐R as it has shown very good reliability in women who have recently given birth (*α* = 0.91) [33] and pregnant women (*α* = 0.91) [34], and has shown high internal consistency when used with men (*α* = 0.96) [35]. We used the suppression sub‐scale of the ERQ as it is a well‐validated measure of emotional suppression showing strong psychometric properties when used in a variety of settings and populations [36, 37, 38]. In addition, it has been used in pregnancy research with studies showing satisfactory/good reliability with pregnant (*α* = 0.82) [39] and postpartum mothers (*α* = 0.73) [40], and has demonstrated measurement invariance when used with men [41].

Responses from each timepoint were scored using the appropriate scoring matrix and entered into a study database. For the EPDS, a threshold of ≥ 14 was applied to mothers which has been found to be a flag to alert clinicians for symptoms of depression [25]. For partners, a two‐point lower score of ≥ 12 was applied as previous work suggests this threshold is a flag to alert clinicians for symptoms of depression in fathers [26]. A GAD‐7 score of ≥ 10 was applied to screen for moderate or severe anxiety [42]. An IES‐R score of ≥ 33 was used to signify the likely presence of PTSD [35]. A threshold for the ERQ suppression scale has not been identified in the literature. As such, we identified the upper quartile range for participants' mean suppression scores (> 2.75 at 23 weeks; > 2.75 at 32 weeks; > 2.50 at 6 weeks postnatal) and applied our own data‐driven threshold value of ≥ 2.75 to identify the proportion of partners and mothers who frequently suppressed their emotions at each time point. Mothers' demographic characteristics and information related to pregnancy history (e.g., type of loss, interpregnancy interval) were obtained from maternity records. Partners were asked to disclose their demographic information (age, gender, ethnicity and marital status) at the beginning of the first questionnaire. Participants who did not complete the first questionnaire were not sent subsequent questionnaires.

### 2.3 Statistical Analysis

Descriptive statistical analysis and comparative analysis was undertaken in SPSS (Version 29) to determine whether there were significant differences in questionnaire scores at different gestations of pregnancy and postnatally. The four measurement scales showed very good internal consistency on all three measurement occasions (Cronbach's alpha > 0.84). Data from partners and mothers were analysed separately. Distribution of data was evaluated by Shapiro–Wilk test. When analysis was restricted to participants who completed all three questionnaires, normally distributed data was analysed using Repeated measures ANOVA. Non‐normally distributed data was analysed using the Friedman test as it is a robust non‐parametric alternative to the Repeated measures ANOVA. Pairwise comparisons or Wilcoxon signed‐rank tests identified significant differences in participants' scores between time points. The Wilcoxon signed‐rank test was used as it is a robust alternative to the paired *t*‐test for comparing two related samples that have non‐normally distributed data. To determine whether there were significant differences in partners' and mothers' group scores across the four measures, normally distributed data was analysed using independent samples *t*‐tests. Non‐normally distributed data was analysed using the Mann–Whitney *U* test. To determine whether both parents within each couple responded in similar ways to the measures, the mean difference between the couple's scores were analysed using a one‐sample *t*‐test.

## 3 Results

**FIGURE 1:**
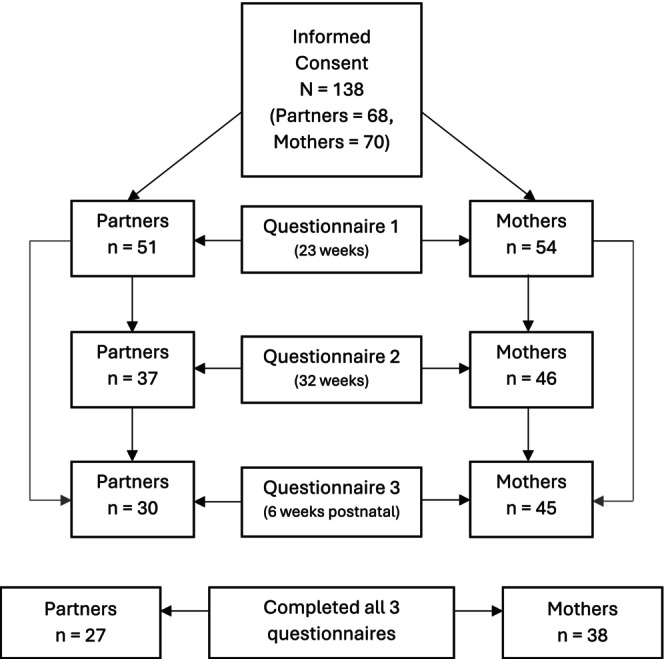
Flow chart showing the number of partners and mothers who gave their informed consent and completed the questionnaire at each time point.

The number of partners and mothers participating in the study at each stage is shown in Figure 1. Fifty‐one partners and 54 mothers completed the questionnaires. Twenty‐seven partners and 38 mothers completed all three questionnaires. One partner identified as non‐binary and all other partners reported their gender as male. All mothers identified as female. Partners and mothers were aged 26–44 (*M* = 35 years; SD = 4.5) and 26–42 (*M* = 35 years; SD = 4.0) respectively when they completed the first questionnaire. The ethnicity, marital status and pregnancy history of participants are summarised in Table 1. Twenty‐five partners and 25 mothers had no living children, and 27 partners and 26 mothers had one or more living children. The number of living children for two of the mothers was unknown. The number of partners and mothers participating in the study at each stage is shown in Figure 1. One couple had a preterm birth before completing the second questionnaire and informed the research team that they did not wish to complete subsequent questionnaires. All other participants who completed the first questionnaire were sent the second and third questionnaires. All participants who remained in the study delivered a live baby between 35 and 41 weeks' gestation.

**TABLE 1:** Ethnicity, marital status and pregnancy history for study participants (*n* = 105).

Demographic characteristic	Partners, *N* (%)	Mothers, *N* (%)
Ethnicity		
White British	47 (92)	42 (78)
White–any other White background	1 (2)	5 (9)
Asian or Asian British–Indian	2 (4)	1 (2)
Asian or Asian British–Pakistani	0 (0)	1 (2)
Asian or Asian British–Any other Asian Background	0 (0)	3 (6)
Black or Black British–African	1 (2)	1 (2)
Mixed–White and Asian	0 (0)	1 (2)
Marital status		
Married	36 (71)	36 (68)
Living with partner	14 (27)	16 (30)
Single/not living with partner	1 (2)	1 (2)
Unknown	0 (0)	1 (2)
Parity		
1	5 (10)	4 (7)
2	27 (53)	26 (48)
≥ 3	16 (31)	21 (39)
Unknown	3 (6)	3 (6)
Interpregnancy interval (months)		
≤ 12	21 (41)	19 (35)
13–36	21 (41)	20 (37)
≥ 37	4 (8)	10 (19)
Unknown	5 (10)	5 (9)
Mode of stillbirth		
Vaginal delivery	42 (82)	45 (84)
Emergency caesarean section	5 (10)	5 (9)
Unknown	4 (8)	4 (7)
Gestation at time of loss (months)		
16–23	22 (43)	21 (39)
24–32	8 (16)	10 (19)
33–41	17 (33)	19 (35)
Unknown	4 (8)	4 (7)
Type of loss		
Second‐trimester loss	15 (29)	14 (26)
Antepartum stillbirth	22 (43)	27 (50)
Termination for medical reasons	7 (14)	6 (11)
Neonatal death	5 (10)	5 (9)
Unknown	2 (4)	2 (4)
Mode of live birth		
Vaginal delivery	14 (27)	14 (26)
Elective caesarean section	20 (39)	21 (39)
Emergency caesarean section	5 (10)	7 (13)
Unknown	12 (24)	12 (22)

### 3.1 Depression

**FIGURE 2:**
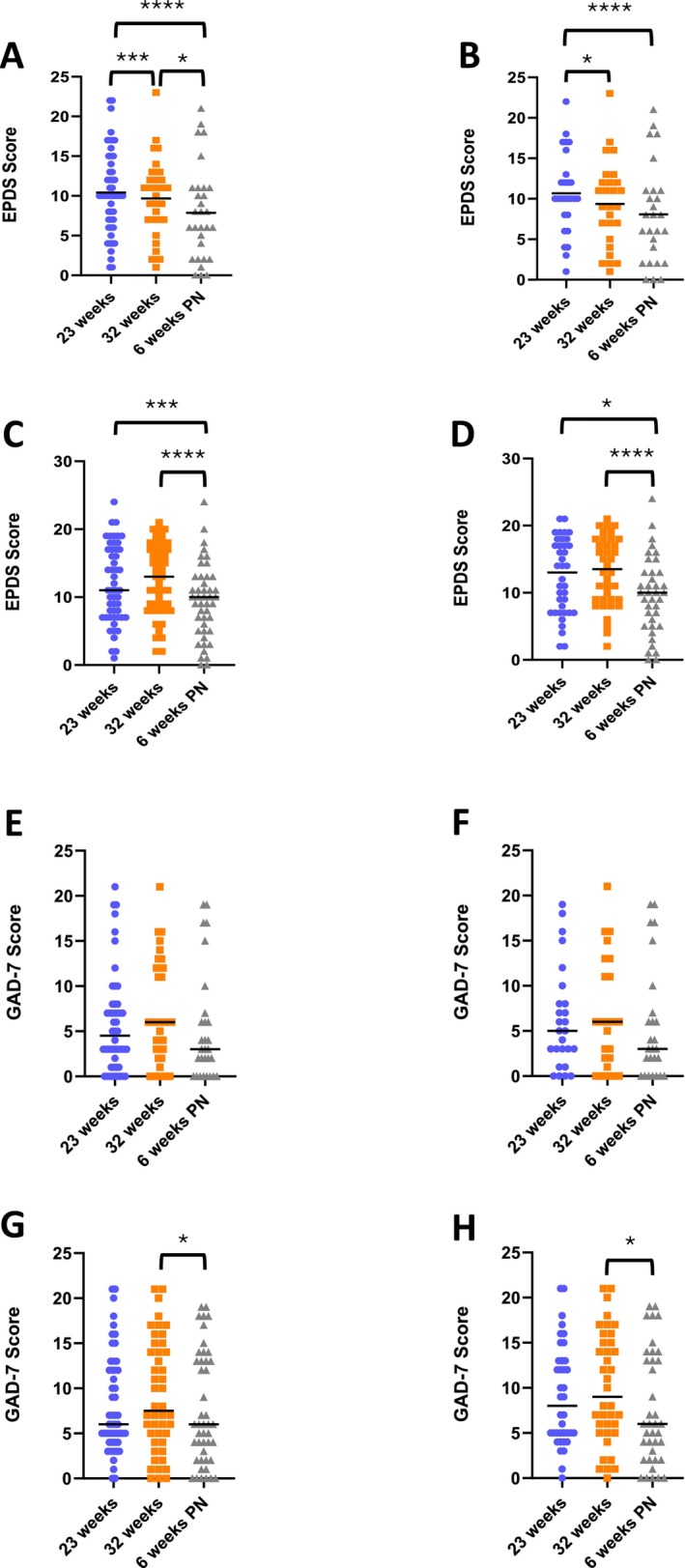
Depression and anxiety as assessed by EPDS and GAD‐7 questionnaires. (A) EPDS at 23 weeks' gestation, 32 weeks' gestation and 6 weeks postnatal for all partners, (B) EPDS at the same gestations for partners who completed all three questionnaires, (C) EPDS completed at 23 weeks' gestation, 32 weeks' gestation and 6 weeks postnatal for all mothers, (D) EPDS completed at the same gestations for mothers who completed all three questionnaires. (E) GAD‐7 scores at 23 weeks' gestation, 32 weeks' gestation and 6 weeks postnatal for all partners, (F) GAD‐7 scores at the same gestations for partners who completed all three questionnaires, (G) GAD‐7 scores completed at 23 weeks' gestation, 32 weeks' gestation and 6 weeks postnatal for all mothers, (H) GAD‐7 scores completed at the same gestations for mothers who completed all three questionnaires. *p < 0.05, ***p < 0.005, ****p < 0.001.

For all non‐birthing partners and partners who completed all three questionnaires, there was a significant reduction in EPDS scores from 23 to 32 weeks' gestation (Figure 2A,B; Tables S1 and S2). In addition, there was a significant reduction in partners' EPDS scores from 32 weeks' gestation to 6 weeks postnatal (Figure 2A; Table S1). However, when analysis was restricted to partners who completed all three questionnaires there was no significant reduction in partners' EPDS scores from 32 weeks' gestation to 6 weeks postnatal (Figure 2B; Table S2). The EPDS demonstrated a significant decline in mothers' scores from 32 weeks' gestation to 6 weeks postnatal but there was no significant change in EPDS scores from 23 to 32 weeks' gestation (Figure 2C; Table S3). The profile of mother's EPDS scores stayed the same when analysis was restricted to mothers who had completed all three questionnaires (Figure 2D; Table S4). Succinctly stated, partners', but not mothers', depression symptoms significantly declined across pregnancy. In addition, partners' and mothers' depression symptoms significantly declined postnatally, however partners' symptoms did not significantly decline when analysis included only those who had completed all three questionnaires. Applying a threshold for the likelihood of major depression (partners' EDPS score ≥ 12; mothers' EPDS score ≥ 14) found 37% of partners and 42% of mothers likely had depression at 23 weeks gestation, 32% of partners and 48% of mothers likely had depression at 32 weeks' gestation, and 17% of partners and 18% of mothers likely had depression at 6 weeks postnatal.

### 3.2 Anxiety

For all partners and partners who returned all three questionnaires, there was no significant change in GAD‐7 scores across any of the time points (Figure 2E,F; Tables S3 and S4). For mothers, the GAD‐7 score showed a significant reduction from 32 weeks' gestation to 6 weeks postnatal, but there was no significant decline in GAD‐7 scores from 23 to 32 weeks' gestation (Figure 2G; Table S3). The profile of GAD‐7 scores was the same for mothers who returned all three questionnaires (Figure 2H; Table S4). To summarise, there were no significant changes in partners' and mothers' anxiety symptoms throughout pregnancy; mothers, but not partners, anxiety symptoms significantly declined postnatally. Applying a threshold for significant (moderate or severe) anxiety (GAD score ≥ 10) found 20% of partners and 37% of mothers had significant anxiety at 23 weeks' gestation, 32% of partners and 46% of mothers had significant anxiety at 32 weeks' gestation, and 21% of partners and 36% of mothers had significant anxiety at 6 weeks postnatal.

### 3.3 Post‐Traumatic Stress

**FIGURE 3:**
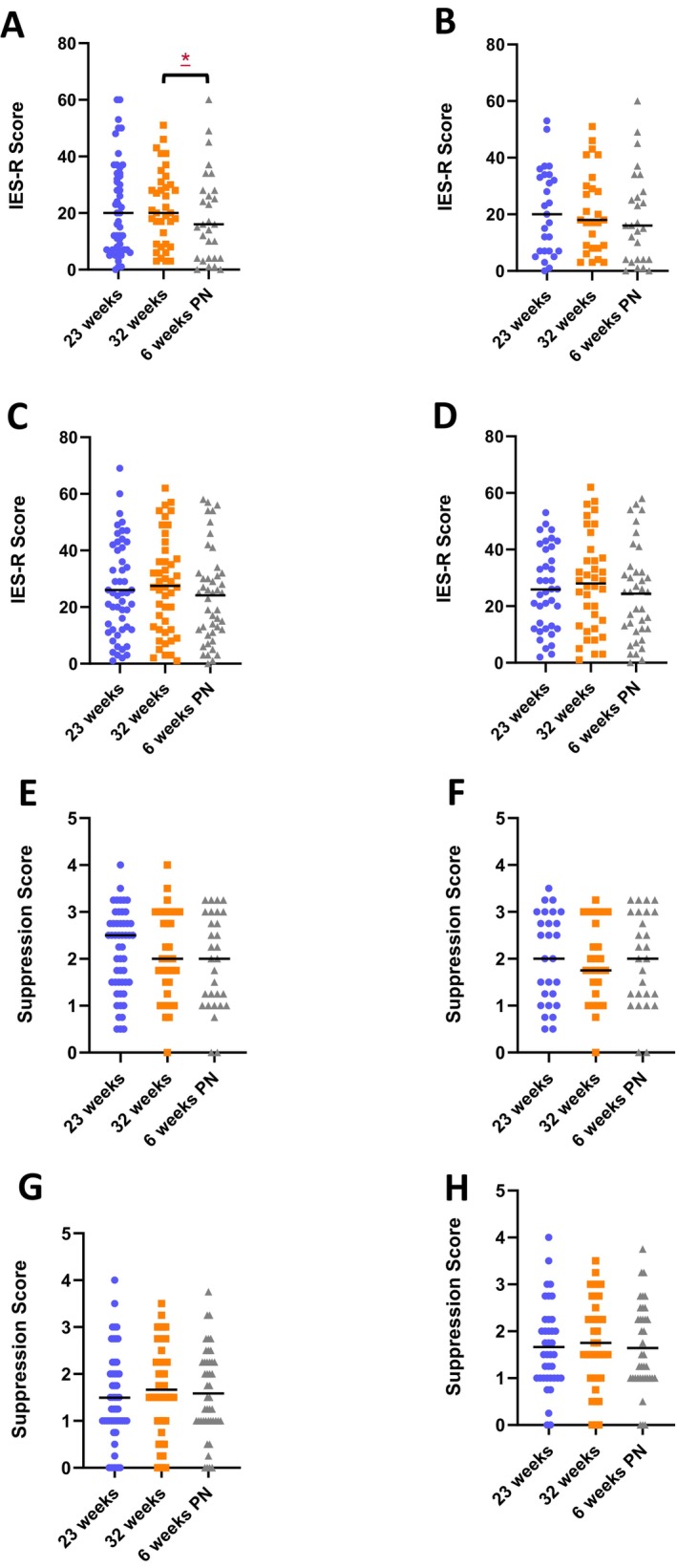
Post‐traumatic stress and Emotional Suppression assessed by the IES‐R and ERQ questionnaires. (A) IES‐R at 23 weeks' gestation, 32 weeks' gestation and 6 weeks postnatal for all partners, (B) IES‐R at the same gestations for partners who completed all three questionnaires, (C) IES‐R completed at 23 weeks' gestation, 32 weeks' gestation and 6 weeks postnatal for all mothers, (D) IES‐R completed at the same gestations for mothers who completed all three questionnaires, (E) Suppression at 23 weeks' gestation, 32 weeks' gestation and 6 weeks postnatal for all partners, (F) Suppression at the same gestations for partners who completed all three questionnaires, (G) Suppression completed at 23 weeks' gestation, 32 weeks' gestation and 6 weeks postnatal for all mothers, (H) Suppression completed at the same gestations for mothers who completed all three questionnaires.

For partners, there was no significant change in IES‐R scores across any of the time points for all partners and partners who completed all three questionnaires (Figure 3A; Tables S3 and S4). For mothers, there was no significant change in IES‐R scores across any of the time points (Figure 3C; Table S1). The profile of mother's IES‐R scores was the same when analysis was restricted to mothers who returned all questionnaires (Figure 3D; Table S2). Succinctly stated, partners', and mothers' PTS symptoms did not significantly change throughout pregnancy or after the birth of a live baby. Applying a threshold for the likelihood of PTSD (IES‐R score ≥ 33) found 28% of partners and 35% of mothers likely had PTSD at 23 weeks' gestation, 22% of partners and 35% of mothers likely had PTSD at 32 weeks' gestation, and 21% of partners and 22% of mothers likely had PTSD at 6 weeks postnatal.

### 3.4 Emotional Suppression

There was no significant change in partners' or mothers' suppression scores across any of the time points (Figure 3E,G; Tables S1 and S3). This was the same when analysis was restricted to partners and mothers who completed all three questionnaires (Figure 3F,H; Tables S2 and S4). Applying a threshold for the frequent use of emotional suppression (≥ 2.75) found 35% of partners and 18% of mothers frequently suppressed their emotions at 23 weeks' gestation, 38% of partners and 22% of mothers frequently supressed their emotions at 32 weeks' gestation, and 33% of partners and 13% of mothers suppressed their emotions frequently at 6 weeks postnatal.

### 3.5 Correlations

Relations between depression, anxiety, PTS and suppression scores are reported in Table S5. The nature of the correlation altered depending upon the factor being assessed and sampling time point; however, in general, depression, anxiety and PTS were positively correlated antenatally and postnatally. Emotional suppression showed a greater number of positive correlations with other measures in the antenatal period.

### 3.6 Between‐Group Comparison of Partners' and Mothers' Psychological Distress and Emotional Suppression

At 23 weeks, partners had lower GAD‐7 scores (*z* = −2.54, *p* = 0.011) and higher emotional suppression scores when compared to mothers (*z* = 3.35, *p* < 0.001). At 32 weeks, partners had a lower EPDS score (*M* = 9.7; SD = 4.7) compared to mothers (*M* = 12.5; SD = 5.3; *p* = 0.01) and lower GAD‐7 scores when compared to mothers (*z* = −1.96, *p* = 0.05). There were no significant differences between partners' and mothers' scores of psychological distress and emotional suppression at any other time point.

### 3.7 Within‐Couple Comparison Between Partners' and Mothers' Psychological Distress and Emotional Suppression

The within‐couple analysis (see Table S6) shows that there was a significant difference between partners' and mothers' within‐couple score for anxiety and emotional suppression at 23 weeks' gestation, depression and emotional suppression at 32 weeks' gestation, and PTS at 6 weeks postnatal; the results indicate that within the couple the partner had lower anxiety at 23 weeks, depression at 32 weeks, and PTS at 6 weeks postnatal compared to the mother, and higher emotional suppression compared to the mother at 23 and 32 weeks. All other differences did not reach statistical significance indicating that levels of depression and PTS at 23 weeks, anxiety and PTS at 32 weeks, and depression, anxiety and emotional suppression at 6 weeks postnatal do not differ significantly within couples.

## 4 Discussion

The current study described partners' and mothers' antenatal and postnatal symptoms of depression, anxiety, PTS and emotional suppression in a pregnancy after perinatal death. Our study is one of the first to describe bereaved partners' symptoms of psychological distress and emotional suppression in a subsequent pregnancy.

### 4.1 Comparison With Previous Literature

Depression symptoms for partners agree with previous studies showing these are highest in early pregnancy and decrease as the pregnancy progresses and into the postnatal period [13, 16]. In addition, previous studies have reported average EPDS scores of 10.8 and 9.2 in the third trimester with 28% of mothers scoring above threshold [13, 43]; this is comparable with the average EDPS scores in this study for partners at 9.7% and 32% scoring above threshold at 32 weeks' gestation. Our study shows higher rates of symptoms of postnatal depression for partners who have experienced perinatal loss (17% in the postnatal period) compared to 8%–10% of male partners in the wider population [44, 45]. Depression scores for mothers were higher than our earlier work [13] with 18% of mothers scoring above threshold postnatally compared to 11% previously. Partners' anxiety levels did not fall after the birth of a live baby, which contrasts with findings from mothers in this study and previous studies which showed a reduction in symptoms postnatally [16, 17]. Mothers' anxiety levels in this study were also higher than previous work: 46% and 36% of mothers scored above the threshold for anxiety at 32 weeks' gestation and 6 weeks postnatal compared to 27% and 23% of mothers in our previous study [13].

Previous work found that 15.6% and 18.8% of partners had a diagnosis of PTSD (current/lifetime) in the antenatal period in a pregnancy following a loss [16] our results show higher rates, with 28% and 22% of partners likely suffering from PTSD in the second and third trimesters, and approximately 1 in 5 parents experiencing PTSD after the birth of a live baby. The results generally indicate that partners' and mothers' symptoms of psychological distress are likely to be concurrent (i.e., if a partner has symptoms of PTS at 23 weeks' gestation, the mother will also have symptoms of PTS at that gestation). This supports previous work which shows positive associations of mental health disorders between companions [46, 47]. Our study is the first to quantify parents' emotional suppression in a pregnancy following loss. However, our findings support qualitative work in which non‐birthing partners describe taking on the role of the supporter to the mother after pregnancy loss, often disregarding and hiding their own feelings to support her [9, 10].

### 4.2 Interpretation of Key Findings

Incidences of postnatal depression in mothers have increased from 10% to 16% to 24% in 2014, 2018 and 2020, respectively [48]. The proportion of mothers experiencing symptoms of postnatal depression since the pandemic is unknown. However, one explanation for why mothers reported increased symptoms of postnatal depression in our study (compared to 2016–18) may be that the COVID‐19 lockdowns and subsequent social isolation have increased the prevalence of symptoms of postnatal depression in mothers nationwide. It may also be that some of our participants experienced baby loss during the pandemic, which meant they had a lack of social support, thus contributing to negative mental health.

Partners may be continuing to experience high anxiety levels postnatally as these may be related to the health and safety of their newborn baby [49], and mothers may also be increasingly anxious postnatally about their baby contracting or dying from an illness or disease such as COVID‐19. Indeed, mothers interviewed in 2023–24 for our qualitative study reported anxieties related to the safety and health of their baby postnatally [50]. The negative publicity about UK maternity care, with half of hospital maternity departments in England being rated as inadequate or requiring improvement [51], may also be contributing to mothers' anxiety related to the birth.

Pregnancy loss and/or a traumatic birth can be a trigger for the onset of PTSD [52]. PTS symptoms in our study did not change across the course of a pregnancy and are likely to continue into the postnatal period for mothers, which indicates that PTS symptoms are unlikely to improve without psychological intervention. More research is needed to determine whether PTS symptoms reduce for partners postnatally. We propose that partners more frequently suppress their emotions than mothers because many partners feel that they must support the mother; thus, they feel that they cannot confide in her for emotional support. Indeed, in our qualitative interviews, many partners said it was their role to support the mother; however, they would have liked support from a healthcare professional or another partner with shared experience to discuss their feelings [49].

Partners' symptoms of psychological distress may negatively impact on mothers' mental health, or mothers' symptoms may negatively impact on partners' mental health; this may indicate that if one person in the couple is psychologically distressed then it may negatively impact their partner's well‐being, potentially precipitating a cycle of distress. As such, supporting the mental health of one person in the couple is likely to indirectly benefit their partner. However, there were some differences observed (between groups and within‐couple) between partners and mothers for some measures of psychological distress (e.g., depression at 32 weeks) and emotional suppression (e.g., at 23 weeks) suggesting that partners and mothers may have unique psychological support needs at different time points during a pregnancy following a loss.

### 4.3 Implications for Clinical Practice

There are currently no specific care pathways for partners who are experiencing a pregnancy following a perinatal death. Our findings show that partners may need specialist support, particularly in the early stages of pregnancy and continuing into the postnatal period. Specific care pathways would need to be developed for partners, as obstetric services typically have no signposting or referral procedures currently in place to support them. In addition, partners would likely benefit from being signposted by healthcare professionals to postnatal support groups if such groups were available within their local area. Partners likely hide their emotions more than mothers, particularly during pregnancy; thus, they would benefit from opportunities to have 1‐to‐1 discussions with a trusted healthcare professional at maternity care appointments. In our qualitative interviews, partners expressed they would like discussions with healthcare professionals separate from the mother, as they would feel more able to openly discuss their feelings and concerns [49].

Mothers' psychological distress reported in this study is much higher than our previous work suggesting the urgent need for psychological support for this group both during and after pregnancy. Partners and mothers should be screened (e.g., by using standardised screening tools [53]) and monitored throughout a subsequent pregnancy for symptoms of psychological distress, and offered counselling or other appropriate psychological support. It would be optimal for screening to begin in early pregnancy (e.g., 8–10 weeks) and repeated in the second and third trimesters of pregnancy, and postnatally, as psychological distress symptoms are likely to begin in early pregnancy and remain at elevated levels.

Minimising parents' psychological distress would also benefit babies and children as prenatal maternal distress is associated with increased risk of mental health disorders and atypical neurodevelopment in offspring [54]. Parents should also be monitored and given support in the postnatal period to minimise psychological distress and to promote positive parent–infant relationships with their new baby. Parents are also likely to benefit from being signposted to peer support non‐profit organisations such as Branch Baby Loss Network [55] (UK based) where they can connect via WhatsApp with other parents who are experiencing pregnancy following perinatal death. Training and support should be given to healthcare professionals delivering care to parents who have experienced a perinatal death to ensure parents are supported [56] as parents value well‐trained and caring staff to emotionally support them after baby loss [57, 58].

### 4.4 Strengths and Limitations

This study contributes to the literature by evaluating both parents' psychological distress and emotional suppression in a pregnancy following a perinatal loss. However, there are limitations that can be considered and used to suggest directions for future research. First, participants were recruited from one specialist clinic in the North‐West of England; therefore, we cannot assume that findings can be generalised to all parents who have experienced a pregnancy following a loss. Indeed, it is likely that bereaved parents who do not access specialist care are experiencing even higher rates of psychological distress than those reported in this study. It may also be that parents who have higher rates of psychological distress are at increased risk of self‐harm. Future studies could consider recruiting participants from a larger number of hospitals and clinics to extend findings and consider investigating whether parents who are experiencing a pregnancy following baby loss have an increased risk of self‐harm than parents who have not experienced baby loss.

Second, we did not include a control group; therefore, we cannot make comparisons between parents who have and have not experienced perinatal loss. Further studies could consider recruiting parents with no previous history of loss to compare groups; this would also enable comparison of emotional suppression scores to determine whether partners who have lost a baby are hiding their emotions during pregnancy more than partners who have not lost a baby. In addition, some participants who initially gave informed consent did not complete the questionnaires; it may be that parents who did not complete the questionnaire had higher levels of psychological distress and emotional suppression than parents who participated. Indeed, emotional distress may be a barrier to research participation, as it has been shown to be a factor influencing decision‐making after stillbirth [59]. As such, the impact of perinatal loss on parents' psychological wellbeing and suppression in a subsequent pregnancy may be underestimated in this sample.

Thirdly, one partner reported their gender as non‐binary. To ensure that their identity was not disclosed, we did not conduct or report analysis that would have investigated whether psychological distress in parents may vary by gender. It would be important for future studies to aim to recruit a more gender‐diverse sample to identify if levels of psychological distress differ in gender‐diverse people, and to adequately represent gender‐diverse identities in pregnancy after loss research. In addition, future studies could consider asking participants to report their sexual orientation to be inclusive of non‐heterosexual couples and to identify the role of sexual orientation in the psychological distress of parents in a pregnancy after perinatal death.

Fourthly, participants' demographic information related to their pregnancy history was collected retrospectively (after the data had been analysed) when participants' questionnaire data had already been anonymised. As such, it was not possible to match this demographic information to participants' questionnaire data; thus, we could not explore how these variables might correlate with our measures. However, exploring how these factors might be related to levels of psychological distress (e.g., whether a longer interpregnancy interval is associated with lower levels of PTSD) would be an important avenue for future research and would provide greater insight into the factors which contribute to increased symptoms of anxiety, depression and PTS for bereaved parents in a subsequent pregnancy.

Lastly, 75% of partners and 77% of mothers in our study who gave their informed consent completed the first questionnaire. However, many partners in our study were lost to follow‐up: only 54% and 44% of partners who gave their informed consent completed the second and third questionnaires, respectively, compared to 66% and 64% of mothers. Partners may have been more likely to complete the first questionnaire as it was sent shortly after their Rainbow clinic appointment where they discussed the study with a trusted healthcare professional. However, when parents completed the questionnaire for the second or third time, they likely hadn't had recent contact with the specialist antenatal care team; this is more likely for partners compared to mothers as mothers typically have more frequent communication with the team by phone or email. As such, this distance from the healthcare professionals may have contributed to loss to follow‐up, particularly for partners. Future studies might consider ways in which healthcare professionals or researchers can facilitate more frequent contact with partners throughout the duration of the study (e.g., a phone call before administering subsequent questionnaires) to increase retention rates. In addition, it was not possible to conclude whether partners' depression or PTS symptoms changed after the birth of a live baby as the *p*‐values were close to or at significance. Further research which retains a greater number of partners for postnatal participation would be needed to identify if symptoms change after birth.

## 5 Conclusion

This study demonstrates that partners and mothers who have experienced a perinatal loss are likely to experience high levels of psychological distress in a subsequent pregnancy, which may continue into the postnatal period. Specialist mental health support offered in conjunction with antenatal and postnatal care in a dedicated pregnancy after loss service is one way to support parents. Further research is needed with minority ethnic groups, gender‐diverse persons and non‐heterosexual couples to determine if findings extend to these groups. Intervention research is needed to determine which types of support are most beneficial for improving psychological outcomes.

## Author Contributions

A.E.P.H., D.S. and J.B. contributed to the study design. A.E.P.H. obtained funding and had overall responsibility for the study. E.B., R.B., E.T. and A.E.P.H. were responsible for recruiting participants and collecting demographic data. J.B. analysed the data with input from A.E.P.H. and D.S. A.E.P.H., D.S. and J.B. were responsible for the drafting of the manuscript. All authors gave approval for the final version of the manuscript.

## Ethics Statement

The study was granted ethical approval by the Health Research Authority, South Central–Berkshire B Research Ethics Committee (Ref 22/SC/0108).

## Conflicts of Interest

The authors declare no conflicts of interest.

## Supporting information


**Table S1.** Pairwise Comparisons for Partners’ Depression, and Mothers’ PTS and Suppression.
**Table S2**. Pairwise comparisons from Repeated Measures ANOVAs for Partners’ Depression, and Mothers’ PTS and Suppression.

**Table S3.** Wilcoxon Signed‐Rank Tests for Partners’ Anxiety, PTS and Suppression, and Mothers’ Depression, Anxiety and Suppression.

**Table S4.** Friedman Tests and Post Hoc Comparisons for Partners’ Anxiety, PTS and Suppression, and Mothers’ Depression, Anxiety and Suppression.

**Table S5.** Bivariate Correlations Between Depression, Anxiety, PTS, and Emotional Suppression.

**Table S6.** Within‐Couple Analysis for Depression, Anxiety, PTS, and Emotional Suppression.

## Data Availability

Due to the sensitive nature of the study, participants were not asked to give permission for data sharing. Anonymised, unlinked data are available from the authors on request.
