# Supplementary material for: Psychological Distress, Post‐Traumatic Stress and Emotional Suppression in a Pregnancy After a Perinatal Death: A Longitudinal Survey
Source: BJOG. 2025 May 13;132(10):1469–80. doi: 10.1111/1471-0528.18212 (PMC12315086; doi:10.1111/1471-0528.18212)
Supplement: Supplementary file 1 — Table S1. Pairwise Comparisons for Partners’ Depression, and Mothers’ PTS and Suppression. Table S2. Pairwise comparisons from Repeated Measures ANOVAs for Partners’ Depression, and Mothers’ PTS and Suppression. [file BJO-132-1469-s001.docx]

| **Table S1.** Pairwise Comparisons for Partners’ Depression, and Mothers’ PTS and Suppression | | | | | | | | | | | | |
| --- | --- | --- | --- | --- | --- | --- | --- | --- | --- | --- | --- | --- |
|  |  |  | Time 1 | | | Time 2 | |  |  | 95% Cl | | Cohen’s *d* |
|  | Variable | N | *M* | | *SD* | *M* | *SD* | *t*(df) | *p* | *LL* | *UL* |  |
| Partner | EPDS | 37 | 11.32 | | 5.35 | 9.68 | 4.68 | 3.09(36) | 0.004 | 0.57 | 2.73 | 3.24 |
| Mother | IES-R | 44 | 25.52 | | 15.20 | 26.73 | 16.80 | -0.78(43) | 0.439 | -4.31 | 1.91 | 10.23 |
|  | SUP | 44 | 1.56 | | 0.96 | 1.65 | 0.99 | -1.11(43) | 0.270 | -0.25 | 0.07 | 0.54 |
|  |  |  | Time 2 | | | Time 3 | |  |  | 95% Cl | | Cohen’s *d* |
|  | Variable |  | *M* | | *SD* | *M* | *SD* | *t*(df) | *p* | *LL* | *UL* |  |
| Partner | EPDS | 27 | 9.37 | | 5.30 | 8.07 | 6.05 | 2.07(26) | 0.049 | 0.01 | 2.58 | 3.26 |
| Mother | IES-R | 39 | 28.18 | 16.70 | | 24.46 | 16.45 | 1.89(38) | 0.067 | -0.27 | 7.71 | 12.30 |
|  | SUP | 39 | 1.76 | 0.95 | | 1.65 | 0.92 | 0.913(38) | 0.367 | -0.12 | 0.33 | 0.70 |

| **Table S2.** Pairwise comparisons from Repeated Measures ANOVAs for Partners’ Depression, and Mothers’ PTS and Suppression | | | | | | | | | | | |
| --- | --- | --- | --- | --- | --- | --- | --- | --- | --- | --- | --- |
|  |  |  | Time 1 | | Time 2 | |  |  | 95% CI | | *p* |
|  | Variable | N | *M* | *SD* | *M* | *SD* | Mean Difference | Standard Error | *LL* | *UL* |  |
| Partner | EPDS | 27 | 10.67 | 4.99 | 9.37 | 5.30 | 1.296 | 0.455 | 0.13 | 2.46 | 0.026 |
|  |  |  |  |  |  |  |  |  |  |  |  |
| Mother | IES-R | 38 | 25.92 | 14.52 | 28.00 | 16.89 | -2.079 | 1.651 | -6.22 | 2.06 | 0.647 |
|  | SUP | 38 | 1.66 | 0.91 | 1.75 | 0.96 | -0.086 | 0.092 | -0.32 | 0.15 | 1.000 |
|  |  |  | Time 2 | | Time 3 | |  |  | 95% CI | | *p* |
|  | Variable | N | *M* | *SD* | *M* | *SD* | Mean Difference | Standard Error | *LL* | *UL* |  |
| Partner | EPDS | 27 | 9.37 | 5.30 | 8.07 | 6.05 | 1.296 | 0.627 | -0.31 | 2.90 | 0.146 |
|  |  |  |  | |  |  |  |  |  |  |  |
| Mother | IES-R | 38 | 28.00 | 16.89 | 24.42 | 16.67 | 3.579 | 2.017 | -1.48 | 8.64 | 0.253 |
|  | SUP | 38 | 1.75 | 0.96 | 1.64 | 0.93 | 0.105 | 0.115 | -0.18 | 0.40 | 1.000 |
| *Note.* A Bonferroni adjustment for multiple comparisons was applied. | | | | | | | | | | | |
