# Supplementary material for: Psychological Distress, Post‐Traumatic Stress and Emotional Suppression in a Pregnancy After a Perinatal Death: A Longitudinal Survey
Source: BJOG. 2025 May 13;132(10):1469–80. doi: 10.1111/1471-0528.18212 (PMC12315086; doi:10.1111/1471-0528.18212)
Supplement: Supplementary file 2 — Table S3. Wilcoxon Signed‐Rank Tests for Partners’ Anxiety, PTS and Suppression, and Mothers’ Depression, Anxiety and Suppression. [file BJO-132-1469-s005.docx]

| **Table S3.** Wilcoxon Signed-Rank Tests for Partners’ Anxiety, PTS and Suppression, and Mothers’ Depression, Anxiety and Suppression | | | | | | | | |
| --- | --- | --- | --- | --- | --- | --- | --- | --- |
|  | Measure | Time | N | Ranks | Mean Rank | Sum of Rank | *z* | *p* |
| Partner | GAD | 1-2 | 37 | Negative Ranks | 16.03 | 256.50 | -0.167 | 0.867 |
|  |  |  |  | Positive Ranks | 15.97 | 239.50 |  |  |
|  |  | 2-3 | 27 | Negative Ranks | 10.06 | 90.50 | -1.167 | 0.243 |
|  |  |  |  | Positive Ranks | 6.50 | 45.50 |  |  |
|  | IES-R | 1-2 | 37 | Negative Ranks | 21.53 | 323.00 | -0.157 | 0.875 |
|  |  |  |  | Positive Ranks | 16.33 | 343.00 |  |  |
|  |  | 2-3 | 27 | Negative Ranks | 13.29 | 252.00 | -1.958 | 0.050 |
|  |  |  |  | Positive Ranks | 14.07 | 98.50 |  |  |
|  | SUP | 1-2 | 37 | Negative Ranks | 15.64 | 219.00 | -0.369 | 0.712 |
|  |  |  |  | Positive Ranks | 13.36 | 187.00 |  |  |
|  |  | 2-3 | 27 | Negative Ranks | 10.79 | 75.50 | -0.442 | 0.658 |
|  |  |  |  | Positive Ranks | 8.68 | 95.50 |  |  |
| Mother | EPDS | 1-2 | 44 | Negative Ranks | 16.81 | 269.00 | -1.483 | 0.138 |
|  |  |  |  | Positive Ranks | 21.45 | 472.00 |  |  |
|  |  | 2-3 | 39 | Negative Ranks | 19.75 | 553.00 | -3.905 | <0.001 |
|  |  |  |  | Positive Ranks | 11.00 | 77.00 |  |  |
|  | GAD | 1-2 | 44 | Negative Ranks | 17.44 | 296.50 | -0.576 | 0.565 |
|  |  |  |  | Positive Ranks | 19.45 | 369.50 |  |  |
|  |  | 2-3 | 39 | Negative Ranks | 20.07 | 542.00 | -2.493 | 0.013 |
|  |  |  |  | Positive Ranks | 18.09 | 199.00 |  |  |
|  | SUP | 1-2 | 44 | Negative Ranks | 16.10 | 161.00 | -1.235 | 0.217 |
|  |  |  |  | Positive Ranks | 14.42 | 274.00 |  |  |
|  |  | 2-3 | 39 | Negative Ranks | 17.00 | 289.00 | -0.814 | 0.416 |
|  |  |  |  | Positive Ranks | 14.79 | 207.00 |  |  |
