# Supplementary material for: Psychological Distress, Post‐Traumatic Stress and Emotional Suppression in a Pregnancy After a Perinatal Death: A Longitudinal Survey
Source: BJOG. 2025 May 13;132(10):1469–80. doi: 10.1111/1471-0528.18212 (PMC12315086; doi:10.1111/1471-0528.18212)
Supplement: Supplementary file 3 — Table S4. Friedman Tests and Post Hoc Comparisons for Partners’ Anxiety, PTS and Suppression, and Mothers’ Depression, Anxiety and Suppression. [file BJO-132-1469-s002.docx]

| **Table S4.** Friedman Tests and Post-Hoc Comparisons for Partners’ Anxiety, PTS and Suppression, and Mothers’ Depression, Anxiety and Suppression | | | | | | | | | | |
| --- | --- | --- | --- | --- | --- | --- | --- | --- | --- | --- |
|  |  | N | Chi-square | df | *p* |  |  |  |  |  |
| Partner | GAD | 27 | 1.04 | 2 | 0.596 |  |  |  |  |  |
|  | IES-R | 27 | 5.91 | 2 | 0.052 |  |  |  |  |  |
|  | SUP | 27 | 1.11 | 2 | 0.573 |  |  |  |  |  |
|  |  |  |  |  |  |  |  | Post-Hoc Comparisons | | |
|  |  | N | Chi-square | df | *p* |  |  | T-Stat | Std. Error | *p* |
| Mother | EPDS | 38 | 15.01 | 2 | <0.001 |  | Time 1-2 | -0.21 | 0.23 | 0.359 |
|  |  |  |  |  |  |  | Time 2-3 | 0.82 | 0.23 | <0.001 |
|  | GAD | 38 | 6.91 | 2 | 0.032 |  | Time 1-2 | -0.25 | 0.23 | 0.276 |
|  |  |  |  |  |  |  | Time 2-3 | 0.58 | 0.23 | 0.012 |
|  | SUP | 38 | 1.79 | 2 | 0.408 |  |  |  |  |  |
