# Supplementary material for: Psychological Distress, Post‐Traumatic Stress and Emotional Suppression in a Pregnancy After a Perinatal Death: A Longitudinal Survey
Source: BJOG. 2025 May 13;132(10):1469–80. doi: 10.1111/1471-0528.18212 (PMC12315086; doi:10.1111/1471-0528.18212)
Supplement: Supplementary file 4 — Table S5. Bivariate Correlations Between Depression, Anxiety, PTS, and Emotional Suppression. [file BJO-132-1469-s003.docx]

**Table S5.** Bivariate Correlations Between Depression, Anxiety, PTS, and Emotional Suppression

|  | 1 | 2 | 3 | 4 | 5 | 6 | 7 | 8 | 9 | 10 | 11 | 12 |
| --- | --- | --- | --- | --- | --- | --- | --- | --- | --- | --- | --- | --- |
| 1. T1 Depression | — | .81*** | .70*** | .15 | .81*** | .74*** | .72*** | .22 | .68*** | .69*** | .61*** | .13 |
| 2. T1 Anxiety |  | — | .72*** | .22* | .69*** | .73*** | .66*** | .23* | .59*** | .71*** | .56*** | .16 |
| 3. T1 PTS |  |  | — | .28** | .62*** | .56*** | .76*** | .27* | .49*** | .54*** | .55*** | .18 |
| 4. T1 Suppression |  |  |  | — | .19 | .20 | .24* | .81*** | .08 | .15 | .19 | .70*** |
| 5. T2 Depression |  |  |  |  | — | .81*** | .74*** | .24* | .71*** | .68*** | .57*** | .11 |
| 6. T2 Anxiety |  |  |  |  |  | — | .76*** | .30** | .56*** | .69*** | .55*** | .13 |
| 7. T2 PTS |  |  |  |  |  |  | — | .34** | .56*** | .56*** | .73*** | .20 |
| 8. T2 Suppression |  |  |  |  |  |  |  | — | .09 | .12 | .31* | .79*** |
| 9. T3 Depression |  |  |  |  |  |  |  |  | — | .80*** | .69*** | .15 |
| 10. T3 Anxiety |  |  |  |  |  |  |  |  |  | — | — | .12 |
| 11. T3 PTS |  |  |  |  |  |  |  |  |  |  | — | .34** |
| 12. T3 Suppression |  |  |  |  |  |  |  |  |  |  |  | — |
| *Note.* T = time.  **p* <.05. ***p* <.01. ****p* <. 001. | | | | | | | | | | | |  |
