# Supplementary material for: Psychological Distress, Post‐Traumatic Stress and Emotional Suppression in a Pregnancy After a Perinatal Death: A Longitudinal Survey
Source: BJOG. 2025 May 13;132(10):1469–80. doi: 10.1111/1471-0528.18212 (PMC12315086; doi:10.1111/1471-0528.18212)
Supplement: Supplementary file 5 — Table S6. Within‐Couple Analysis for Depression, Anxiety, PTS, and Emotional Suppression. [file BJO-132-1469-s004.docx]

**Table S6.** Within-Couple Analysis for Depression, Anxiety, PTS, and Emotional Suppression

|  | Couples (n) | Mean difference | Std. Error Difference | *t* | df | *p* |
| --- | --- | --- | --- | --- | --- | --- |
| 1. T1 Depression | 42 | -1.452 | 1.080 | -1.345 | 41 | 0.186 |
| 2. T1 Anxiety | 40 | -2.500 | 1.231 | -2.031 | 39 | 0.049 |
| 3. T1 PTS | 40 | -3.275 | 2.990 | -1.095 | 39 | 0.280 |
| 4. T1 Suppression | 41 | 0.665 | .177 | 3.752 | 40 | <0.001 |
| 5. T2 Depression | 28 | -2.607 | 1.139 | -2.289 | 27 | 0.030 |
| 6. T2 Anxiety | 28 | -2.643 | 1.502 | -1.760 | 27 | 0.090 |
| 7. T2 PTS | 28 | -3.929 | 3.293 | -1.193 | 27 | 0.243 |
| 8. T2 Suppression | 28 | 0.464 | .211 | 2.203 | 27 | 0.036 |
| 9. T3 Depression | 22 | -1.182 | 1.650 | -.716 | 21 | 0.482 |
| 10. T3 Anxiety | 21 | -2.714 | 1.860 | -1.460 | 20 | 0.160 |
| 11. T3 PTS | 21 | -8.762 | 3.748 | -2.338 | 20 | 0.030 |
| 12. T3 Suppression | 22 | 0.216 | .262 | .823 | 21 | 0.420 |

*Note*. T = Time. The mean difference is equal to the partners’ scores minus the mothers’ scores. Negative values indicate higher scores for the mothers
